# Pathogenesis of *ETV6/RUNX1*-positive childhood acute lymphoblastic leukemia and mechanisms underlying its relapse

**DOI:** 10.18632/oncotarget.16367

**Published:** 2017-03-18

**Authors:** Congcong Sun, Lixian Chang, Xiaofan Zhu

**Affiliations:** ^1^ Center for Pediatric Blood Disease, State Key Laboratory of Experimental Hematology, Institute of Hematology and Blood Diseases Hospital, Chinese Academy of Medical Sciences, and Peking Union Medical College, Tianjin, P.R. China

**Keywords:** ETV6/RUNX1, childhood acute lymphoblastic leukemia, mechanisms, initiation, relapse

## Abstract

*ETV6/RUNX1* (*E/R*) is the most common fusion gene in childhood acute lymphoblastic leukemia (ALL). Multiple lines of evidence imply a “two-hit” model for the molecular pathogenesis of *E/R*-positive ALL, whereby *E/R* rearrangement is followed by a series of secondary mutations that trigger overt leukemia. The cellular framework in which *E/R* arises and the maintenance of a pre-leukemic condition by *E/R* are fundamental to the mechanism that underlies leukemogenesis. Accordingly, a variety of studies have focused on the relationship between the clones giving rise to the primary and recurrent *E/R*-positive ALL. We review here the most recent insights into the pathogenic mechanisms underlying *E/R*-positive ALL, as well as the molecular abnormalities prevailing at relapse.

## INTRODUCTION

Acute lymphoblastic leukemia (ALL), a malignancy characterized by clonal proliferation of arrested immature blood cell precursors (blasts), progresses rapidly and often appears without an obvious prodrome [1]. It can be classified according to either immunophenotype or by molecular identification of a growing number of somatic genetic alterations characterized during the last decades [2, 3]. In pediatric B-cell acute lymphoblastic leukemia (ALL), the most frequent chromosomal lesion is t(12;21)(p13;q22), which results in its molecular genetic counterpart, the *ETV6/RUNX1* (also known as *TEL/AML1*) fusion gene [4, 5]. This alteration occurs in approximately 25% of childhood ALL diagnosed between the ages of 2 and 10 years, with a median age of 4 years [6–8]. Based on the excellent molecular response to treatment and beneficial clinical outcome, it was originally believed that this rearrangement is a rather favorable prognostic indicator [9, 10]. However, this notion was subsequently disputed as others found predominantly late relapses occurring in up to 20% of patients [7, 11]. Thus, the prognostic implication of the *ETV6/RUNX1 (E/R)* fusion continues to be debated. Over the past decades, remarkable advances have been made on the precise role of the *E/R* fusion gene product in childhood ALL. We review here some of the major findings that highlight the impact of the *E/R* fusion gene on childhood ALL at initiation and relapse.

## PATHOGENESIS OF ETV6/RUNX1-POSITIVE CHILDHOOD ALL

The *E/R* fusion gene develops mainly as an early, prenatal event in childhood ALL. The expression of *E/R* results in the generation of a persistent pre-leukemic clone, which postnatally converts, at low frequency, to ALL after the acquisition of necessary secondary genetic abnormalities [12, 13]. Several studies have addressed the mechanism by which *E/R* contributes to leukemogenesis, including the necessary secondary genetic lesions, the cellular framework in which *E/R* initially arises and the maintenance of a pre-leukemic condition.

### *ETV6/RUNX1*-positive childhood ALL: a “two-hit” disease

**Figure 1:**
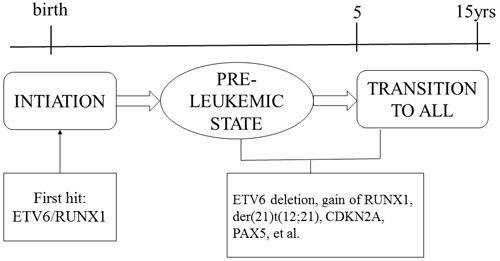
Two-hit model of the natural history of E/R-positive ALL

In considering the natural history and etiology of childhood ALL, a key problem is when and how the *E/R* fusion gene is generated and whether this is an early or initiating event [14]. The detection of the *E/R* fusion sequence in identical twins and in neonatal blood spots of children with ALL indicate that this gene fusion originates in the prenatal period [14–21]. A concordance rate for ALL of only 5% in twin children, the highly variable postnatal latency period (up to 14 years) of the disease, and evidence from transgenic animal models of E/R fusion all suggest that additional postnatal genetic events are required for the development of clinically overt leukemia [22–30]. Thus, although the *E/R* fusion gene constitutes the first step in the transformation of normal cells into malignant cells, a modest number of recurrent or “driver” copy number alterations (CNAs) are most likely necessary for leukemia to develop (Figure 1).

Initially, most studies addressing secondary leukemia-promoting genetic changes have focused on the deletion of the normal, or non-rearranged, *ETV6* allele [31]. This genetic abnormality accounts for approximately 70% of t(12;21)-positive ALL cases [32]. *ETV6* can function as a tumor suppressor [33] and dimerize with *E/R* to reduce its transforming activity [34]. The other common genetic changes in the *ETV6* and *RUNX1* genes are an extra *RUNX1* gene (23%) and/or an extra der(21)t(12;21) (10%) [35, 36]. Approximately 20% of the t(12;21)-positive patients have more than one additional genetic change in *ETV6* or *RUNX1* [35, 37]. These genomic changes are so prevalent that their existence can be taken as significant circumstantial evidence for a cryptic *E/R* fusion in pediatric ALL.

However, to fully understand the genetic evolution of *E/R*-positive ALL, identification of the entire spectrum of genetic changes that accompany this fusion gene is necessary. The genome of *E/R*-positive ALL has been well characterized at the copy number and cytogenetic level. A large number of secondary genetic abnormalities in *E/R*-positive ALL may be observed by conventional or molecular cytogenetics methods (Table 1) [38–48]. Each *E/R*-positive ALL patient has an average of 3.5 additional CNAs (range 0-14). Among these, several are recurrent deletions in genes controlling B-cell lineage differentiation, cell cycle, or nuclear hormone response [38, 47, 49]. In addition, the majority of *E/R*-positive cases display a unique pattern of CNAs, indicating that a large heterogeneity exists within this ALL subtype [47]. The frequent larger alterations include deletions of 12p, 6q, 9p, 11q, and 13q, losses of X, 8, and 13, and gains of 21, 4, 10, and 16 [38, 44, 50]. The deletion of 12p (39%) is the most common abnormality, its most likely pathogenetically important outcome being the loss of wild-type *ETV6* allele [51]. The 9p deletion can be seen in up to 25% of *E/R*-positive patients, and it often affects the tumor suppressor *CDKN2A/CDKN2B* and the B-cell differentiation regulator *PAX5* [35, 38, 40]. Genome-wide, high resolution CNAs analyses have revealed an increasing number of putative causative submicroscopic changes in diverse genes such as *CD200, BTLA, TBL1XR1, GLRX2, NR3C2, NR3C1, TCF3, EBF1, LEF1, IKZF1, CCNC, ARMC2, BTG1, SPANXB, TP63, PTPRJ, ATF7IP*, and *MGA* [13, 36, 42, 47, 52]. In addition, a rare case of an *E/R*-positive ALL patient that acquired the *BCR-ABL1* gene rearrangement as a secondary change has been recently reported [53]. The genes listed above belong to many different classes and affect various pathways. For instance, the loss of *PAX5, EBF1* and *TCF3* can lead to impaired production of mature B-cells [49]; deletions of *CD200* and *BTLA* contribute to a disrupted function of the immune system [47]; deletion of *TBL1XR1*, which is a key component of the *SMRT* and *N-CoR* compressor complexes, is associated with overexpression of genetic targets of the retinoic acid and thyroid hormone receptors [42]. Several studies have depicted the relationship between CNAs and their assumed temporal order of appearance with branching and distance-based oncogenetic trees [47, 54]. These tree models showed that the *CDKN2A/B, ETV6*, del(6q), *PAX5* and +16 are probably among the earliest aberrations [47]. But further studies are required to confirm the sequential order of acquisition for different CNAs that can be observed directly.

**Table 1:** Common (>5% frequency) secondary genetic changes observed in E/R-positive ALL

Chr	Genetic abnormality	Frequency (%)	Possible candidate genes
**12**	del(12p) del(12)(p11-13) del(12)(p13.2) del(12)(p21.3)	18-69	ETV6, CDKN1B, BCL2L14, BTG1
**6**	del(6q) del(6)(q21-27) der(6)t(X;6)	13-37	AIM1, PRDM1, FOXO3, CCNC, FYN
**9**	del(9p) del(9)(p13-24) del(9)(p21.3) del(9)(p13.2)	7-29	CDKN2A/B, PAX5, MTAP, JAK2, P14ARF, P16INK4a/ARF
**21**	*gain(21) gain(21q)	10-25	RUNX1
**5**	del(5)(q31.3-33.3)	23	NR3C1, EBF1
**14**	del(14)(q32.33) del(14)(q11.2)	3-21	DPF3
**3**	del(3)(p21) del(3)(q26.32) del(3)(q13.2) del(3)(p14.2)	3-21	LIMD1, ARPP-21, ULK4, FHIT, CD200, BTLA, TBL1XR1
**7**	del(7)(q34) del(7)(p14.1)	6-18	IKZF1
**4**	gain(4q) del(4)(q31.23)	6-17	NR3C2, YIPF7, ARHGAP10
**11**	del(11q) del(11)(q22-23) del(11)(q23-25) del(11)(p13)	6-13	CD44, RAG1, RAG2, BACL2, GNG3, HNRPUL2, TTC9C, ATM, KMT2A
**19**	del(19)(q13.11) del(19)(q13.32)	6-13	CEBPA, UHRF1, GRLF1, NPAS1, TMEM160
**22**	del(22)(q11.22)	13	-
**X**	loss(X)(in females) gain(Xq)(in males) gain(Xp)	4-11	SPANXB, HMGB3, FAM50A, HTATSF1
**13**	del(13q) del(13)(q14-34) del(13)(q12.2)	5-10	BTG1, RB1, SERP2, DLEU2, STBP4, TRIM13, KCNRG, MIRN16-1, MIRN15A, DLEU1, DLEU7
**1**	del(1)(q31.3)	10	TROVE2, GLRX2, CDC73, B3GALT2, PDE4B
**15**	del(15)(q15.1) del(15)(q26.1)	10	LTK, MIRN626
**2**	del(2)(p25.3)	9	-
**8**	del(8p) del(8)(p11-23)	6-8	TOX
**16**	gain (16)	6	-

Chr, chromosome;

*Additional copy of chromosome 21 or an additional copy of the der(21)t(12;21)(p13;q22)

These secondary genetic events are major rate-limiting events during the process of formation of *E/R*-positive ALL and may be expected to drive the evolution of overt leukemic cells, culminating in a clinical diagnosis of ALL. However, the mechanisms triggering these second hits have not been completely elucidated. A study reported that the expression of *E/R* was associated with increased ROS levels [55]. Elevated ROS could increase genetic instability and double-strand DNA breaks [56], thus leading to the accumulation of secondary mutations. Recently, Papaemmanuil et al. used exome and low-coverage whole-genome sequencing to characterize secondary events associated with leukemic transformation, confirming that aberrant RAG recombinase activity contributes critically to secondary events leading to leukemic transformation in *E/R*-positive ALL [13].

### Clonal origin of *ETV6/RUNX1*-positive childhood ALL

Understanding the pathogenesis of *E/R*-positive ALL requires knowledge of not only the specific contributory fusion and the secondary genetic hits but also the cellular framework in which they arise and function. Most of the leukemic cells in *E/R*-positive ALL can be characterized by phenotypes (CD19+, CD10+, TdT+ and RAG1/2+) and genotypes (ongoing clonal *IgH* rearrangements) indicative of a B precursor cell identity. However, the cell of origin in which the initial functional impact of the fusion gene occurs has been contentious. It may lie anywhere antecedent to B precursor cells in the lineage hierarchy [57]. *IgH, TCR* and *ETV6/RUNX1* are leukemia-specific molecular markers in *E/R*-positive ALL. Analysis of these markers could illuminate when *ETV6/RUNX1* gene fusion occurs during B cell development.

Confirmation of the leukemic cell of origin is fundamental to identify the cellular and molecular mechanisms that underlie leukemogenesis. A popular hypothesis is that completely transformed cancer clones are settled in a hierarchical pattern and sustained by a few “cancer stem cells” [58]. However, less is known about the “precancerous” cells upon which first-hit events occur [25]. In fact, it has been reported in other leukemias that the initiating events could occur upstream within the hematopoiesis hierarchy. For instance, early transformation events have been demonstrated to take place in CD34+/CD38− cells of Philadelphia chromosome-positive ALL [59]. In *E/R*-positive ALL, this fusion transcript can occur during B-cell development before rearrangement of *IgH* and *TCR* genes [60]. These rearrangements are features of B-cell precursor leukemia and commence at the pro-B cell stage of normal B-cell lineage development [61]. Thus, the *E/R* fusion premalignant clone may occur before the pro-B cell stage. Sequence analysis of the *E/R* fusion region further supports this possibility, as there are no binding motifs indicative of RAG1/2 or TdT activity [62, 63]. In accord with this, expansion of early multipotent progenitors has been detected after retroviral expression of the *E/R* fusion gene [64]. Besides, modeling data from murine experiments have also suggested a possible multipotential progenitor origin of the fusion gene [26, 65]. The activity of *E/R* in early progenitors or hematopoietic stem cells (HSCs) may account for the expression of the myeloid markers CD13 and CD33 in up to 48% of E/R-positive ALL patients [60]. The explanation for *E/R*'s requirement to be expressed in leukemic stem and/or progenitor cells is that lymphoid progenitors lack self-renewal and cannot live long enough to accumulate the mutations required for full transformation [26]. It is advantageous for *E/R*-positive pre-leukemic cells to preserve the regulatory control mechanisms of normal stem cells to persist in their niche and avoid exhaustion.

However, studies on clonal *IgH* and/or *TCR* gene rearrangements in *E/R*-positive ALL have demonstrated that the fetal cell type in which clonal advantage is elicited by *E/R* can be propagated after B-lineage commitment [25, 57, 66]. Consistent with this conclusion, retroviral transduction of the *E/R* fusion gene in murine and human models suggested that *E/R* could promote self-renewal of early B-cell precursors [25, 66]. Human modeling data showed that a small portion of CD34+CD38–/^low^CD19+ cells were present in *E/R*-positive ALL patients [25, 67]. This kind of cell population, which may function as pre-leukemic stem cells, has significant self-renewal potential and contains a mixture of pro-B cell- and HSC–associated gene expression profiles [25]. Recently, a study analyzed all varieties of *Ig* and *TCR* gene rearrangements in five pairs of monozygotic twins concordant for ALL. They proved that the *E/R* fusion initially expanded in an early B-cell lineage-committed progenitor, most likely at the pro-B, or possibly at the pre-B, cell stage [57]. The effect of *E/R* at the pre-B or pro-B cell stage is intriguing, as it may prove that the *E/R* fusion gene can directly influence the phenotype of leukemic cells.

Understanding the cellular targets of primary mutations and the ensuing malignant transformation is fundamental to understanding the function of the *E/R* fusion. In addition, this is also relevant to cancer therapy, where specific targeting of tumor-propagating cells may be desirable. Thus, it seems safe to conclude that the jury is still out on this issue, and further research is still required.

### *ETV6/RUNX1* contribution to the pre-leukemic and leukemic states

Observations on normal cord blood and monozygotic twins demonstrated that the *E/R* fusion gene induces a pre-leukemic phenotype that can remain covert for up to 15 years [12]. Therefore, *E/R* is not only responsible for the initiation of leukemia but also essential for disease progression and maintenance. Although the expression of *E/R* interferes with critical regulatory functions [68], maintenance of a pre-leukemic condition by *E/R* fusion itself is not sufficient to explain the evolutionary and clinical emergence of ALL [69]. Instead, additional selective pressure and clonal advantage have been demonstrated to participate in this process [12, 70, 71].

Both *ETV6* and *RUNX1* are known regulators of hematopoiesis. The *E/R* fusion gene includes the N-terminal pointed (PNT) dimerization domain of *ETV6* and the N-terminal Runt homology domain (RHD) and the C-terminal transactivation (TA) domain of *RUNX1* [35]. The resulting hybrid fusion protein has been shown to maintain transcription factor properties [72]. It can bind to *RUNX1* target genes and potentially convert *RUNX1* to a negative transcriptional regulator [73, 74]. Maximal repression requires the binding of mSin3A, N-CoR and histone deacetylase-3 (HDAC3) to *ETV6* [72]. Although the *E/R* chimeric protein is assumed to act as transcriptional repressor of *RUNX1* target genes, several studies have reported similar levels of gene repression and activation for protein-coding and miRNA genes [68, 71, 75]. In addition, the *E/R* fusion gene product can disrupt the transcriptional repression of wild-type *ETV6* by dimerization via the PNT domain [76].

**Figure 2:**
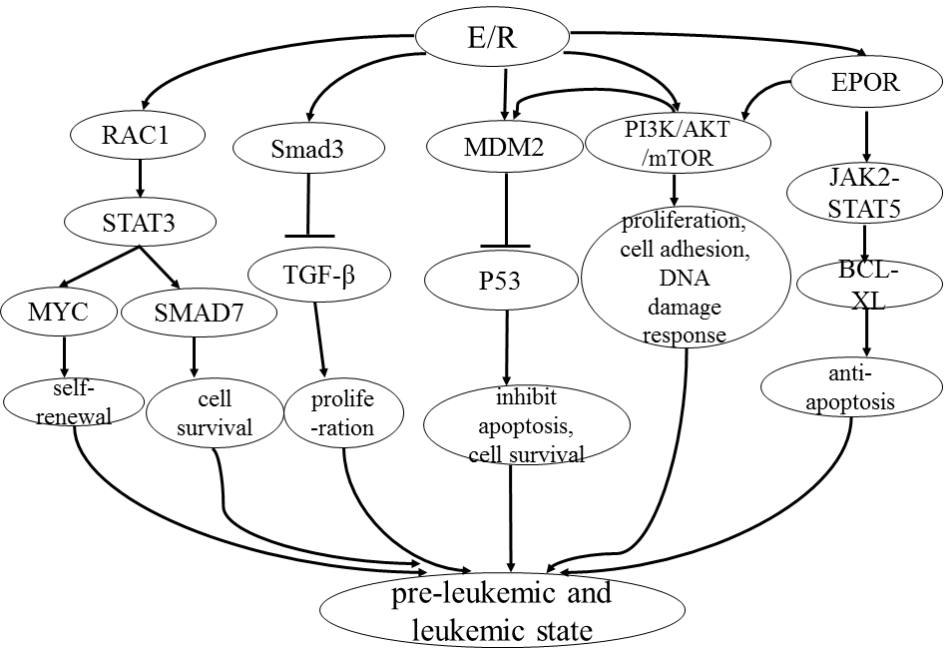
Model for pathways influenced by E/R 1) E/R regulates the phosphorylation of STAT3 via activation of RAC1, resulting in the transcriptional induction of MYC. 2) E/R can bind to a principal TGF-β signaling target, Smad3, which has an inhibitory impact on the response to TGF-β signaling. 3) E/R can upregulate MDM2 promoter activity; MDM2 functions as a crucial negative regulator of p53, repressing its activity. 4) E/R activates PI3K/AKT/mTOR signaling and interferes with proliferation, cell adhesion and DNA damage response. Activation of PI3K/AKT/mTOR signaling also promotes the phosphorylation of MDM2 and its localization in the nucleus where it binds to p53. 5) E/R can directly bind the EPOR promoter and increase cell survival through activation of the JAK2-STAT5 pathway and upregulation of antiapoptotic BCL-XL. EPOR is also one of the upstream signaling components of the PI3K/AKT/mTOR signaling cascade. All these signal pathways facilitate the E/R pre-leukemic state.

Although the *E/R* rearrangement involves two well-characterized transcription factors, the molecular pathways affected by the translocation are also responsible for leukemia clone maintenance and propagation (Figure 2). First of all, *E/R* can reduce sensitivity to the inhibition of proliferation mediated by *TGF-β* signaling, suggesting that the dysregulated immune responses to infection might promote the malignant evolution of pre-leukemic clones [70]. The *E/R* fusion gene can also induce signal transducer and activator of transcription 3 (*STAT3*) activation, aiding to the self-renewal and leukemogenic activity of leukemia cells [77, 78]. *STAT3* activation has been shown to transcriptionally induce *MYC* [77] and *SMAD7* [78] gene expression in *E/R* leukemic cells. In addition, the *E/R* gene product can activate the phosphoinositide 3-kinase (PI3K)/AKT/mammalian target of rapamycin (mTOR) signaling pathway [79], resulting in proliferation and cell survival of leukemic cells. Reflecting the importance of PI3K/AKT/mTOR signaling cascade in many cellular tasks, several PI3K/AKT/mTOR-regulated processes, including cell adhesion and DNA damage response, are also affected in *E/R*-positive leukemia [79]. Moreover, *E/R* can directly bind to the EPOR promoter and increase cell survival through activation of the JAK2-STAT5-BCL-XL pathway [12] and the PI3K/AKT/mTOR pathway [80]. Last but not least, the chimeric E/R protein can upregulate *MDM2* promoter activity. *MDM2* functions as a crucial negative regulator of *P53*, and ultimately represses *P53* signaling [68, 81]. The suppression of *P53* signaling is a crucial event in the development of leukemic cell survival [81].

Epigenetic alterations, including DNA methylation and histone modifications, have also been reported to contribute to ALL progression [82, 83]. Stephan et al. combined methylome and transcriptome approaches to identify epigenetic biomarkers specific for *E/R*-positive ALL, and showed the effects of methylation on the expression of 17 potential drivers of leukemogenesis [75]. Among these genes, *EPOR* has been shown to have a functional impact on promoting leukemic cell survival [12]. The oncofetal RNA-binding protein *IGF2BP1* (IGF2 mRNA binding protein 1) is another hypomethylated and upregulated gene in *E/R*-positive ALL. As a powerful regulator of *E/R* mRNA stability, *IGF2BP1* may be a prominent player in cell transformation events in *E/R*-associated leukemogenesis [84–86]. Several miRNA genes are also differentially methylated in ALL patients with the *E/R* fusion gene [75]. *miRNA-320a* was found to be both hypermethylated and downregulated, together with *miRNA-494*, in E/R-positive leukemia [71]. Both miRNAs are coupled to the repression of survivin and their inhibition may promote antiapoptotic signaling. *miRNA200c* is another hypermethylated miRNA gene that was shown to exhibit strong tumor suppressor properties, partially by interacting with the epigenetic regulator and polycomb group protein *BMI1* [87]. In addition, studies on the association between E/R-positive ALL and histone modifications have shown that preserved global histone H4 acetylation [88] and mutations in *NSD2* histone methyltransferase [89] may also promote leukemogenesis. Other miRNA-related mechanisms have also been demonstrated to provide survival advantage to *E/R*-positive leukemia cells. For instance, *hsa-mir-125b-2* is highly expressed in *E/R*-positive leukemia, and can promote cell survival under growth inhibitory and apoptotic conditions in a *P53*-independent manner [90]. In contrast, *mir-181a-1* is significantly reduced in *E/R*-positive cases, and a double-negative loop involving *mir-181a-1* and *E/R* may contribute to *E/R*-driven arrest of differentiation [91].

Recently, a comprehensive analysis of the long non-coding RNA (lnc RNA) transcriptome has been conducted in *E/R*-positive ALL. Both *lnc-RTN4R-1* and lnc-*NKX2-3-1* can be regulated by the E/R fusion protein and might be functionally implicated in leukemogenesis [92]. In addition, *E/R* can downregulate mitotic arrest-deficient 2L1 (*MAD2L1*) to attenuate the mitotic checkpoint (MC) [93]. This may lead to near-triploidy, near-tetraploidy, and trisomy 21 as rather specific types of secondary changes. Furthermore, two recent studies showed a novel cooperative activity of *E/R* with the antiapoptotic protein *BCL2* [94] and the protein tyrosine phosphatase 4A3 (PTP4A3) [95], although the impact on leukemia progression needs to be further explored.

## MECHANISMS OF RELAPSE OF ETV6/RUNX1-POSITIVE CHILDHOOD ALL

Pediatric *ETV6/RUNX1*-positive leukemia generally has favorable long-term remission and survival rates [96], but as in ALL initial manifestation, *ETV6/RUNX1* is the most common structural genetic aberration in childhood B-cell precursor (BCP) ALL at first relapse [97]. Only 10% of *E/R*-positive relapses occur early, and very early relapses are rare. In most patients relapses occur several years after cessation of treatment [98], and occasionally after 10-20 years [99]. Relapsed childhood *ETV6/RUNX1*-positive leukemia is a clinically and biologically heterogeneous disease. A variety of studies have focused on the relationship between the clones giving rise to the primary and recurrent leukemias, as well as the molecular abnormalities responsible for relapse.

### Clonal origins of relapse in childhood *ETV6/RUNX1*-positive ALL

To characterize the clonal origins of *E/R*-positive ALL relapse, many studies have compared *Ig/TCR* rearrangements, genomic boundaries of unrearranged *ETV6* allele, and CNA patterns of matched diagnostic and relapse samples [97, 100–103]. The simplest interpretation of relapse is the re-emergence of progeny derived from the dominant clone observed at diagnosis. This hypothesis was advanced by the discovery that the initial and the relapse clones shared identical clonotypic *Ig/TCR* markers in some cases [104, 105]. Late relapsing cases of ALL are chemosensitive with durable term remissions, possibly implying that initial survival and re-emergence share similar properties [106]. The cells from the original clone might keep smoldering for a long time [107]. Comparison of the genomic boundaries of unrearranged *ETV6* allele indicated that the relapse clone, at least in some patients, derive not from a dominant clone but from a sibling clone present at diagnosis [101–103]. While leukemic clones both at initiation and relapse evolve from a shared ancestral clone [102, 103], the relapse clone seems to be already present at the time of diagnosis at rates of 1×10^−4^ to 5×10^−3^ cells [100]. During initial therapy, this minor population exhibits only moderate reduction with respect to the bulk of diagnostic dominant clones, but rapidly expands before clinical relapse. After relapse development, these initially slow-responding clones can be rapidly eradicated by the relapse treatment, emphasizing their incongruent biology at the two time points of leukemia manifestation [100].

Recently, research on the clonal origins of relapse in childhood ALL in general or *E/R*-positive ALL in particular [108, 109] has drawn the conclusion that, irrespective of time to relapse, relapses can emerge from either major or minor subclones already present at diagnosis [108–110]. These studies mainly compared SNP array–defined CNAs in relapse versus matched diagnosis samples. The relapse-associated CNAs, either present at diagnosis or acquired as new genomic alterations after initial therapy, allow mapping the evolutionary relationship between diagnosis and relapse [101, 109]. In most cases, the clones at diagnosis and relapse have some identical CNAs, indicating a shared clonal origin [109]. Some CNAs exclusively present in relapse are present at low levels at presentation, implying that relapses can indeed originate from a minor subclone at diagnosis [101, 108]. van Delft et al. have devised a scheme to define 4 types of CNAs, and for each of them they indicate possible clonal origins with respect to the matched diagnosis samples [109]. In type 1, the relapse CNAs resemble the CNAs at diagnosis, thus the clonal origins of relapse may be linked to the dominant clone at diagnosis. In type 2, the clone at relapse acquires extra CNAs, and may derive from a derivative minor clone (from the major clone) at diagnosis. In types 3 and 4, if the clone at relapse shows losses and gains of CNAs, or loses all CNAs present at diagnosis and acquires a completely new set of alterations, suggesting that it may derive from a minor clone at diagnosis. Some researchers have equated minor subclones to ancestral clones in a linear clonal succession model [101, 108], whereas recent studies indicate a rather complex, nonlinear, branching clonal structure [30, 109]. In addition, there is no discernable relationship between the size of the subclone at diagnosis and the time to relapse [109].

**Figure 3:**
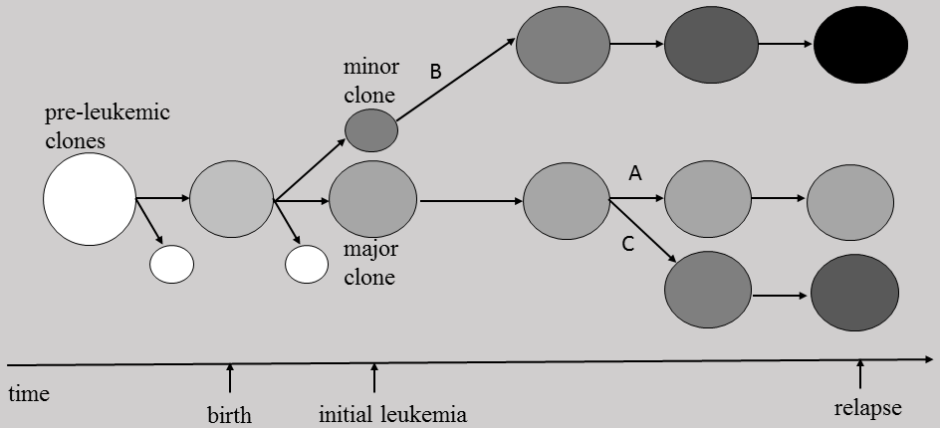
Models for the relapse of ETV6/RUNX1-positive leukemia Circles with different gray shades represent E/R (+) clones. Their respective location within the spectrum designates their pre-leukemic or leukemic nature. The relapse clones originate from either a major or minor clone at presentation. A, the relapse CNAs resemble the CNAs at diagnosis, hence the relapse clone derives from the dominant clone at diagnosis. B, the clone at relapse acquires extra CNAs, thus it derives from a derivative minor clone at diagnosis. C, the clone at relapse acquires a completely new set of CNAs, thus it may derive from a minor clone at diagnosis.

Thus, up to date, it can be concluded that the relapse clone of *E/R*-positive leukemia can originate from either a major or minor clone at presentation (Figure 3). The subclonal diversity at diagnosis provides a variable basis for intraclonal origins of relapse in *E/R*-positive ALL.

### CNAs in *ETV6/RUNX1*-positive ALL relapse

Although the majority of *E/R*-positive relapses can achieve a second continuous complete remission [111], a considerable proportion exhibits drug resistance; these patients eventually experience a subsequent relapse, or even qualify for stem cell transplantation [97]. The clinical heterogeneity among first relapses implies that further genetic abnormalities exist in leukemic cells. Thus, some studies compared diagnostic and relapse CNA patterns to gain further insights into the molecular mechanisms of disease recurrence in *E/R*-positive ALL [97, 108, 112].

A substantial number of CNAs in relapsed *E/R*-positive ALL occur in recurrently affected regions (Table 2) [30, 97, 108, 109, 112]. Also, an increase in the number of CNAs per case has been reported between diagnosis and relapse [101, 108, 109]. The pathways primarily affected by these CNAs are those affecting cell cycle regulation, B-cell development, drug metabolism and drug response [101, 108, 113]. Consistent with the CNAs of *E/R*-positive ALL at diagnosis, chromosomal losses at relapse clearly outnumber gains, and generally involve small genomic regions [97]. Also, additional alterations on chromosomes 12 and 21 are often detected in leukemic cells [36]. The extra der(21)t(12;21) has been reported to be more frequently present in relapsed cases. Patients with this chromosomal change probably have unfavorable long-term clinical outcomes [35]. The recurrent losses at relapse affect chromosomal regions 12p13, 6q21, 6q16.2-3, 8q24, 15q15.1, 9p21, 3p21, 5q, and 3p14.2, whereas gains occur in regions 21q22, 12p and 16 [97, 109]. The genes influenced by loss of 12q13 are *ETV6, BCL2L14*, and *CDKN1B* [97]. Loss of *ETV6* is the most frequent CNA, observed in about 50% of the relapses [36, 97, 108]. Concomitant deletions of the *BCL2L14* and/or *CDKN1B* genes can affect apoptosis and drug response. These lesions are associated with prominently shorter remissions in relapse patients [97, 112]. The combined loss of 12p13 and 6q21 is common in relapse, resulting in co-inactivation of both *FOXO3A* (6q21) and *CDKN1B* (12p13) genes, which may have an adverse prognostic impact [97, 108]. In addition, deletions of *CDKN2A/B*, which are cell cycle regulators, are associated with poor minimal residual disease response and early emerging relapses [112]. Thus, the deletions of *BCL2L14, CDKN1B, CDKN2A/B* and *FOXO3A* may create a genetic background that renders an *E/R*-positive leukemic cell population drug-resistant and favors the development of early relapses. *mir-650* may be associated with time to relapse, as it shows higher expression in early relapse cases [112]. Moreover, several B-lineage transcription factors, among them *PAX5, EBF1*, and *IKZF1*, are associated with B-lymphocyte differentiation and are affected by CNAs in *E/R*-positive relapse as well [97, 108, 113]. CNAs of the X-chromosome and *SLX4IP* show a gender-specific distribution at relapse; gain of Xq and deletion of *SLX4IP* are restricted to male patients [97, 112], while loss of Xq is confined to female patients [97]. In a recent study, Yanliang Jin et al. found that relapses occur more frequently in *E/R*-positive patients who have a three-way translocation pattern, comprising the classic *E/R* translocation plus a fusion of the remaining parts of *ETV6* and *RUNX1* with another chromosome. This pattern might be an important risk factor for relapse in patients with the E/R fusion gene [114].

**Table 2:** Common (>5% frequency) secondary genetic changes observed in E/R-positive relapse ALL

Chr	Genetic abnormality	Frequency (%)	Possible candidate genes
**12**	del(12p) del(12)(q22) del(12)(p13.1-p12) del(12)(p12) del(12)(p13.1) del(12)(p13.3) del(12)(q21.33) gain(12)(p13.2-13.33) gain(12)(p13.33-13.31)	22-67	ETV6, CDKN1B, BCL2L14, BTG1, ATF7IP, VWF, GABARAPL1
**22**	del(22)(q11.22)	61	VPREB1
**9**	gain(9)(p13.3) del(9)(p21.3) del(9)(p13.2)	8-44	CDKN2A/B, PAX5, MTAP, ZCCHC7, OPRS1, IL11RA, CCL27, IFN cluster
**21**	gain(21q) gain(21)(q21.30-22.12)	29-33	RUNX1
**6**	del(6q21)	7-33	FOXO3A, AIM1, ARMC2, SESN1
**11**	del(11p12)	9-32	RAG1, RAG2
**3**	del(3)(p21.21) del(3)(q26.32) del(3)(q13.2) del(3)(p14.2) gain(3)(p21.21)	12-29	FHIT, SMARCC1, MAP4, TBL1XR1, CD200/BTLA, CISH, HEMK1, RASSF1, HYAL2
**20**	del(20)(p12.2) (in males)	26	SLX4IP
**5**	del(5)(q31.3) del(5)(q33.3)	6-21	NR3C1, EBF1
**15**	del(15)(q15.1) del(15)(q14)	10-20	LTK, ITPKA, TY-RO3, BMF
**16**	gain(16) gain(16)(q22.1)	16-19	HSF4, E24F, CTCF, FAM65A
**X**	gain(Xq26.2-28) (in males) gain(Xq27.3-28) (in males)	9-14	SPANX1
**7**	del(7)(q34) del(7)(p12.1-12.2)	7-14	IKZF1, TTC26
**4**	del(4)(q31.23-31.3)	8-14	NR3C2
			
**19**	del(19)(q13.11) del(19)(p13.3)	6-12	GPI, PDCD2L, UBA2, WTIP, TCF3
**13**	del(13q14.2)	10	RB1
**8**	gain(8)(q23.1-24.3)	10	cMYC
**10**	del(10)(q24.1)	10	BLNK(SLP-65)
**2**	del(2)(p21)	7	MSH2

Chr, chromosome;

Glucocorticoids (GC) represent an essential component in frontline and relapse treatment protocols for ALL. GC resistance seems to be associated with ineffective blast cell clearance, disease recurrence and treatment failure in childhood ALL [112, 115]. The GC receptor gene *NR3C1* (5q31.3) encodes the human GC receptor, and its deletion is linked with impaired response to GC [97, 108, 112]. *NR3C1* deletions prevail at relapse and possibly account for the adverse prognosis of some *E/R*-positive relapses [112]. Other deletions associated with GC-mediated apoptosis target the *BCL2* modifying factor (*BMF*) and components of the mismatch repair (MMR) pathways [108]. BMF plays an important role in GC-mediated induction of apoptosis, and its downregulation leads to GC resistance by *E/R*-expressing leukemic cells [108, 116]. Defects in the MMR pathway can alter the apoptotic response to DNA-damaging agents and cause resistance to drugs (e.g. mercaptopurine and prednisone) used in the treatment of childhood ALL [108, 113, 117]. These findings indicate that GC-associated drug resistance may play a role in *E/R*-positive relapse pathogenesis, an observation that may contribute to guide future therapies.

## CONCLUSIONS

The *E/R* fusion gene is the most frequent genetic abnormality in pediatric ALL. Although multiple studies have identified a variety of second genetic hits, their correlation with *E/R* and their effects on the pathogenesis of leukemia need to be further explored. The cell of origin in which the initial functional impact of the *E/R* fusion gene occurs has been contentious. It may lie antecedent to B precursor cells or after B-lineage commitment. Further investigation into the precise cell type of origin has prominent therapeutic significance, as these cells may be the most relevant targets for treatment. In addition, functional studies are needed to unveil the key mechanisms channeling the evolution of *E/R*-positive pre-leukemic cells into B-ALL. *E/R* fusion drives not only the leukemic transformation process but also the maintenance and propagation of leukemia cells. The specific molecular alterations, including components of affected signaling pathways and epigenetic regulators, constitute ideal therapeutic targets. For instance, inhibitions of the aberrantly activated *PI3K/AKT/mTOR* pathway, *STAT3* signaling and/or *MDM2/P53* interaction represent promising therapeutic strategies.

Analyses of CNAs and *Ig/TCR* rearrangements on matched diagnostic and relapse samples showed that relapse in E/R-positive ALL originates from either major or minor clones present at diagnosis. The CNAs specific to relapse contribute to not only the development of relapse but also to drug resistance. GC-associated drug resistance is important in the pathogenesis of E/R-positive ALL relapse. Consideration of the course of childhood ALL implies the need for further biological risk markers that enable a more detailed understanding of poor treatment response. These risk markers may contribute to rationalize specific treatment intensification before stem cell transplantation in poor responding *E/R*-positive relapses.

## Abbreviations

ALL: acute lymphoblastic leukemia
E/R: ETV6/RUNX1
CNAs: copy number alterations
HSC: hematopoietic stem cell
STAT: signal transducer and activator of transcription
IGF2BP1: IGF2 mRNA binding protein 1
MAD2L1: mitotic arrest-deficient 2L1
MC: mitotic checkpoint
GC: glucocorticoids
BMF: Bcl2 modifying factor
MMR: mismatch repair.
